# Expression and diagnostic value of CCT3 and IQGAP3 in hepatocellular carcinoma

**DOI:** 10.1186/s12935-016-0332-3

**Published:** 2016-07-07

**Authors:** E-Na Qian, Shuang-Yin Han, Song-Ze Ding, Xun Lv

**Affiliations:** Department of Gastroenterology and Hepatology, People’s Hospital of Zhengzhou University, No. 7 Wei Wu Road, Zhengzhou, 450003 Henan China

**Keywords:** Hepatocellular carcinoma, Chaperonin containing TCP1 complex subunit 3, IQ-motif-containing GTPase-activating protein-3, α-Fetoprotein, Diagnostic biomarker

## Abstract

**Background:**

To evaluate plasma chaperonin containing TCP1 complex subunit 3 (CCT3) and IQ-motif-containing GTPase-activating protein-3 (IQGAP3) as biomarker for hepatocellular carcinoma (HCC) screening and diagnosis.

**Methods:**

Blood samples were collected from 126 HCC patients with HCC, 88 patients with cirrhosis and 50 healthy volunteers to detect plasma α-fetoprotein (AFP), CCT3 and IQGAP3 levels. Plasma AFP, CCT3 and IQGAP3 protein levels were measured by enzyme linked immunosorbent assay (ELISA).

**Results:**

In the plasma of HCC patients, both CCT3 and IQGAP3 were significantly higher than in patients with cirrhosis and in healthy controls (*P* < 0.01). CCT3 and IQGAP3 protein level correlated well with HCC etiology, tumor size, TNM stage, and child-pugh classification. CCT3 protein had higher sensitivity in the diagnosis of HCC when compared with AFP (87.3 vs 69.8 %). In addition, CCT3 and IQGAP3 protein were able to complement AFP in detecting AFP-negative HCC patients with sensitivity and specificity of 92.1 and 70.5 % and 81.6 and 71.6 %, respectively. In the small HCC group, CCT3 and IQGAP3 protein had sensitivity of 76.6 and 74.5 %, respectively. The combination of AFP + CCT3 + IQGAP3 (0.954) had significantly superior discriminative ability than AFP alone (0.815; *P* < 0.01).

**Conclusions:**

CCT3 and IQGAP3 are novel complementary biomarkers for HCC screening and diagnosis, especially for AFP-negative and small HCC patients.

## Background

Hepatocellular carcinoma (HCC) is the fifth most common cancer in the world and the third leading cause of cancer-related mortality, causing 696,000 deaths each year [1]. Although the prognosis of late-stage HCC has improved during the past two decades, its 5-year survival rate is still low [2].

The most effective therapy for HCC is surgical resection, but tumors at later stages may be inoperable. Traditional detection methods such as ultrasound, magnetic resonance imaging and computed tomography are helpful and efficient in detecting tumor location and metastasis, but cannot reliably detect early-stage HCC, and fail to diagnose HCC when they are used alone [3, 4]. To date, α-fetoprotein (AFP) is the most important serological marker recommended for screening patients at high-risk for HCC. However, nearly 40 % of patients with HCC, including some with small tumors, have normal serological AFP levels [5]. Therefore, it is important to develop reliable biomarkers for early detection and diagnosis of HCC for clinical treatment and prognosis.

The chaperonin containing TCP1 complex (CCT), also called TRiC or c-cpn, mediates protein folding in the cytosol. Chaperonins are ATP-dependent protein-folding machines that are present in all kingdoms of life. They consist of two back-to-back stacked oligomeric rings with a cavity at each end, where protein substrate binding and folding take place [6, 7]. The chaperonin family includes mitochondrial heat shock protein 60, bacterial GroEL, plastid Rubisco subunit-binding protein, and archaea group II chaperonins [8, 9]. CCT3 (60 kDa) is a critical subunit in CCT/TRiC complexes, which plays a significant role in specifically binding these factors during protein folding or refolding. Several studies have shown by quantitative RT-PCR and western blotting that CCT3 is overexpressed in patients with HCC [10–14]. CCT3 can affect the progression of HCC, by having an impact on the transport of phosphorylated signal transducer and activator of transcription (STAT)3/STAT3 into the nuclei of HCC cells. CCT3 may play a role in regulating microtubular structure and function (capture of kinetochores) and affect the cellular sensitivity to these microtubule-targeting agents. However, the role as a biomarker in HCC and other cancer has not been evaluated.

The IQ-motif-containing GTPase-activating protein (IQGAP) family comprises three members: IQGAP1, IQGAP2 and IQGAP3. IQGAP3 is the latest addition to this family. It was involved in the proliferation of epithelial cells, however, its role in tumorigenesis remains to be determined [15, 16]. IQGAP3 promotes cell proliferation through the Ras/extracellular signal-regulated kinase (ERK) pathway [17]. Expression of IQGAP3 was increased during proliferation at sites of cell–cell contact in hepatocytes. Therefore, one of the most important future issues is to identify the cell-density-sensing units at cell–cell contacts in association with IQGAP3. The IQGAP3-related signaling pathway may be one of the complex signaling routes involved in liver regeneration. The contribution of Ras-, Rac-, and Cdc42-binding IQGAP3 to liver regeneration suggests that IQGAP3 might lead to effective coordination of these signaling processes for cell proliferation and tissue remodeling [18].

Skawran et al. [13] found that CCT3 and IQGAP3 genes are all localized on 1q22 and they are up-regulated at the gene level in HCC. To date, it is not clear if CCT3 and IQGAP3 can be detected at the plasma level, nor is the relationship known between plasma AFP, CCT3 and IQGAP3 levels in different stages of HCC. In this study, we investigated CCT3 and IQGAP3 at plasma levels in patients with HCC or cirrhosis and in healthy individuals, and evaluated their application in detecting small and AFP-negative tumors in patients with HCC. Our results demonstrate that CCT3 and IQGAP3 are novel biomarkers complementary to AFP in HCC diagnosis, whose expression is probably independent of AFP. This is especially valuable when AFP is negative and HCC is at an early stage. Thus, determination of CCT3 and IQGAP3 in combination with AFP increases the diagnostic sensitivity and specificity of HCC.

## Methods

### Patient information

We enrolled 126 HCC patients, 88 patients with cirrhosis, and 50 healthy individuals from People’s Hospital of Zhengzhou University (Henan, China) between May 2014 and September 2015. Patients were evaluated and diagnosed by physical examination, laboratory tests, ultrasonography, computed tomography or magnetic resonance imaging. In patients who underwent surgery, HCC was confirmed by pathological examination. No patients received preoperative radiotherapy or chemotherapy and none of the cancer patients had other types of malignancy. HCC was staged according to the child-pugh and tumor-node-metastasis (TNM) classifications [19]. Details about the patients with HCC or cirrhosis are listed in Tables 1 and 2. The Hospital Ethics Committee approved this study. All participants were fully informed and gave written informed consent.

**Table 1:** Clinical characteristics of patients with HCC

Parameters	Patients, n (%)	CCT3	*P* value	IQGAP3	*P* value
		$\overline{\text{x}}$ ± SD, pg/mL		$\overline{\text{x}}$ ± SD, pg/mL	
Age (years)					
≤55	38 (30.2)	285.53 ± 203.38	0.106^a^	217.79 ± 163.06	0.043^a^
>55	88 (69.8)	350.6 ± 207.33		287.83 ± 181.55	
Gender					
Male	84 (66.7)	354.67 ± 217.39	0.08^a^	270.35 ± 167.19	0.748^a^
Female	42 (33.3)	283.6 ± 199.51		259.43 ± 201.11	
Etiology					
HBV(+)	72 (57.1)	284.79 ± 195.82	0.019^b^	218.82 ± 177.52	0.002^b^
HCV(+)	31 (24.6)	407.16 ± 209.483		342.32 ± 150.01	
Other	23 (18.3)	72.87 ± 209.96		314.70 ± 74.99	
Tumor diameter (cm)					
≤2	47 (37.3)	279.13 ± 217.01	0.011^b^	212.74 ± 170.72	0.025^b^
>2 and ≤5	25 (19.8)	432.92 ± 155.60		327.80 ± 138.07	
>5	54 (42.9)	328.91 ± 205.99		285.39 ± 191.04	
Number of lesions					
≤2	61 (48.4)	272.52 ± 200.87	0.002^a^	230.36 ± 166.22	0.026^a^
>2	65 (51.6)	385.83 ± 199.91		300.82 ± 184.04	
Extra-hepatic metastasis					
Yes	34 (27.0)	407.00 ± 215.60	0.014^a^	334.03 ± 207.84	0.27^a^
No	92 (73.0)	302.88 ± 198.33		241.83 ± 160.53	
Differentiation degree					
Well	47 (37.3)	316.21 ± 208.54	0.245^b^	252.85 ± 154.02	0.133^b^
Moderate	43 (34.1)	308.37 ± 201.41		235.53 ± 178.15	
Poor	36 (28.6)	377.25 ± 211.73		322.03 ± 199.74	
TNM stage					
I + II	71 (56.3)	282.56 ± 199.01	0.003^a^	232.5 ± 158.43	0.017^a^
III + IV	55 (43.7)	393.47 ± 203.17		310.85 ± 194.09	
Child-pugh classification					
A	51 (40.4)	265.75 ± 209.34	0.006^b^	203.08 ± 164.94	0.000^b^
B	33 (26.3)	400.58 ± 199.30		357.45 ± 149.83	
C	42 (33.3)	355.50 ± 192.4		272.67 ± 186.31	

*HBV* hepatics B virus, *HCV* hepatics C virus, *HCC* hepatocellular carcinoma, *SD* standard deviation, *TNM* tumor-node-metastasis

^a^By Mann–Whitney U test

^b^By Kruskal–Wallis test

**Table 2:** CCT3 and IQGAP3 protein level in different patient groups

Groups	Patients (*n*)	CCT3 mean ± SD, pg/mL	*P* value	IQGAP3 mean ± SD, pg/mL	*P* value
Total HCC	126	330.98 ± 207.51	<0.01*	266.71 ± 178.47	<0.01*
Cirrhosis	88	82.03 ± 96.53	<0.05**	66.44 ± 63.97	>0.05^#^
Healthy controls	50	50.34 ± 27.27	<0.01***	59.50 ± 51.08	<0.01***
AFP-negative HCC	38	337.66 ± 202.55	<0.01*	261.05 ± 171.20	<0.01*
Small HCC	47	279.13 ± 217.02	<0.01*	212.74 ± 170.73	<0.01*
AFP-negative small HCC	27	306.56 ± 1196.48	<0.01*	232.41 ± 149.13	<0.01*

* *P* < 0.05 (vs. cirrhosis)

** *P* < 0.05 (vs. healthy controls

*** *P* < 0.05 (vs. HCC)

^#^ *P* > 0.05 (vs. healthy controls)

### Blood sampling

Peripheral blood samples (3–5 mL) from each patient were collected in anti-coagulated tubes containing ethylene diamine tetraacetie acid (EDTA). All blood samples from patients with HCC were collected before surgery. The samples from healthy donors were obtained from the Hospital Physical Examination Center. Samples were centrifuged at 1000×*g* for 15 min at room temperature, and stored at −80 °C until testing.

### Measurement of plasma AFP, CCT3 and IQGAP3 protein levels

Plasma CCT3 and IQGAP3 (sensitivity, 5.0 pg/mL; assay range 31.2–1000 pg/mL) levels were measured using a commercially available ELISA kit (Novatein Biosciences, Woburn, MA, USA). Plasma AFP levels were detected using an ELISA kit (R&D Systems, Minneapolis, MN, USA) (sensitivity, 0.046 ng/mL; assay range 0.312–20.0 ng/mL). Ten microliters of plasma samples were mixed with 40 μL of sample dilution buffer and incubated in 96-well plates coated with antibodies for 30 min at 37 °C. The solutions were decanted followed by washing. Fifty-microliter horseradish-peroxidase-conjugated secondary antibodies were added to the wells and incubated for 30 min. After washing, 50 μL of each chromogen solution A and chromogen solution B were added to the wells, and incubated for 15 min at 37 °C. The reaction was stopped by adding 50 μL stop solution. The OD values were determined in a 96-well plate reader (Bio-Rad Laboratories, Hercules, MA, USA) at 450 nm. All tests were performed in duplicate. Average OD values were calculated and the plasma levels of CCT3, IQGAP3 and AFP proteins were determined by standard curve. Data were collected and analyzed, intra-batch variation was controlled within 5 %, and inter-batch variation was <10 %.

### Statistical analysis

All data are summarized and expressed as the mean ± SD. The Mann–Whitney *U* test or Kruskal–Wallis test was used to compare the distribution of CCT3 and IQGAP3 levels and clinical variables among HCC groups. All *P* values were derived from two-sided tests, and *P* < 0.05 was considered statistically significant. Correlation between CCT3 and IQGAP3 levels was assessed using Spearman correlation test. To compare abilities of tumor markers in diagnosis of HCC, receiver operator characteristic (ROC) curves, which correlate true- and false-positive rates [sensitivity and (1 − specificity)], were constructed using the RocKIT (University of Chicago,USA). A logistic regression model was used to analyze sensitivity and specificity of biomarkers. Additionally, areas under the ROC curve (AUC) were calculated for each marker. The statistical significance of differences between the two AUCs was determined. The optimal cutoff values were calculated using the maximum sum of sensitivity and specificity. Statistical analysis was conducted using SPSS for Windows version 17.0 (SPSS Inc., Chicago, IL, USA).

## Results

### Clinicopathological features of serum CCT3 and IQGAP3 expression in HCC patients

**Fig. 1:**
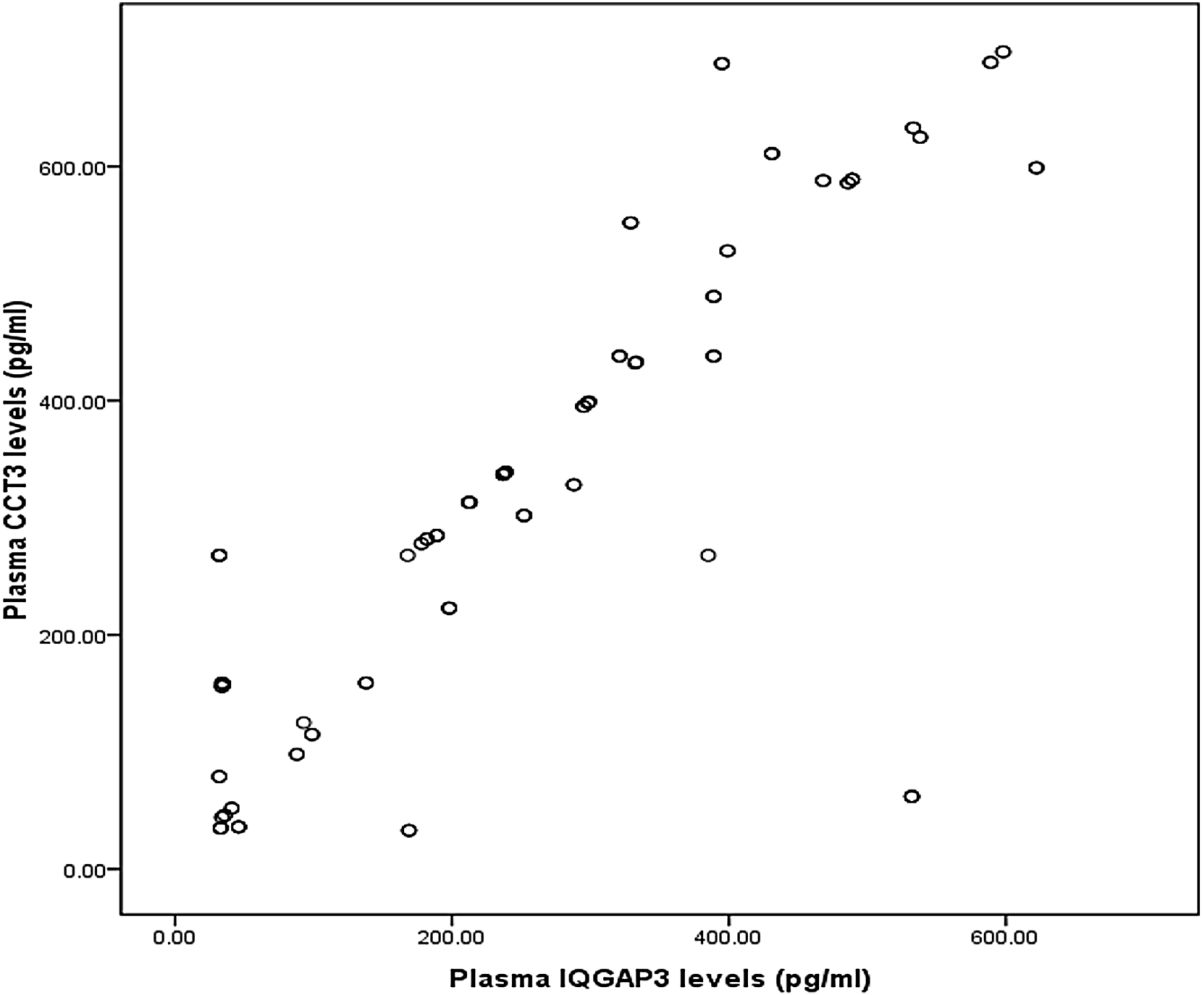
Correlation plasma CCT3 levels with IQGAP3 levels. The correlation of CCT3 level with IQGAP3 level was analyzed in 126 HCC patients. Points show CCT3 or IQGAP3 levels of each of participants, with X axle indicates IQGAP3 levels (pg/mL), and Y axle indicates CCT3 levels (pg/mL)

Clinical characteristics of all 126 HCC patients (84 male and 42 female) were shown in Table 1: 57.1 % had HBV-related hepatitis; 24.6 % had HCV-related hepatitis; 37.3 % had small tumors (single nodule and diameter ≤2 cm); and 30.1 % had AFP-negative HCC (<20.0 ng/mL). The patients in HCC group, 56.3 % were staged as TNM I + II and 43.7 % as III + IV; 40.4 % of patients were classified as child-pugh A, 26.3 % as child-pugh B and 33.3 % as child-pugh C. We found that CCT3 and IQGAP3 protein levels were correlated with etiology of HCC, tumor size, number of cancer nodules and child-pugh classification (*P* < 0.05). Furthermore, (Fig. 1) also indicated that there was correlation between plasma CCT3 and IQGAP3 levels (*r* = 0.824, *P* < 0.01).

### Serum CCT3 and IQGAP3 level in patients with liver diseases and healthy controls

To investigate concentration of serum CCT3 and IQGAP3 in patients with different liver diseases and healthy persons, the levels of CCT3 and IQGAP3 in peripheral blood were detected by ELISA. Expression of serum CCT3 in patients with HCC (330.98 ± 207.51 pg/mL) was significantly higher (*P* < 0.001) than in patients with cirrhosis (82.03 ± 96.53 pg/mL), or healthy controls (50.34 ± 27.27 pg/mL) (Table 2). Similarly, expression of serum IQGAP3 in patients with HCC (266.71 ± 178.47 pg/mL) was evidently higher (*P* < 0.001) than that in patients with cirrhosis (66.44 ± 63.97 pg/mL), or healthy controls (59.50 ± 51.08 pg/mL).

### Plasma CCT3 and IQGAP3 protein levels in patients with small and AFP-negative tumors

**Fig. 2:**
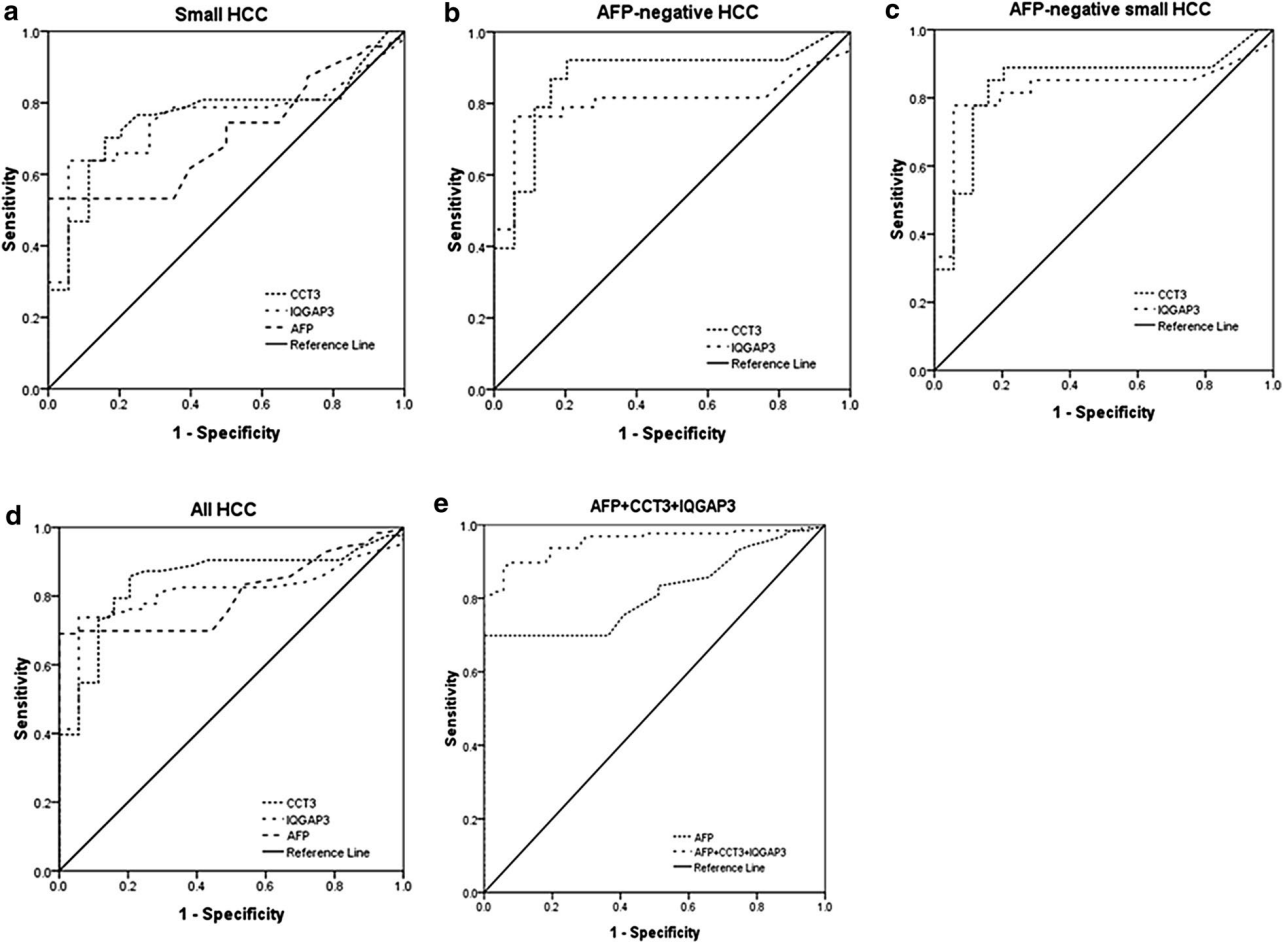
ROC curve of CCT3,IQGAP3 and AFP in HCC, cirrhosis patients. ROC curve were used to analyze the diagnostic performance of CCT3 and IQGAP3 from different groups. The area under the ROC curve (AUC) was shown with 95 % Confidence intervals. **a** ROC curve of CCT3 and IQGAP3 in differentiating small HCC from cirrhosis patients. **b** ROC curve of CCT3 and IQGAP3 in differentiating AFP-negative HCC from cirrhosis patients. **c** ROC curve of CCT3 and IQGAP3 in differentiating AFP-negative small HCC from cirrhosis patients. **d** ROC curve of comparing the accuracy achieved when using AFP, CCT3 and IQGAP3 for diagnosis of HCC. **e** ROC curve of AFP and combined use of AFP, CCT3 and IQGAP3

We compared the levels of CCT3 and IQGAP3 protein in patients with small (*n* = 47) and AFP-negative (*n* = 38) tumors. In the small HCC group, the CCT3 AUC was 0.761, with 95 % Confidence interval (CI) 0.663–0.860, and IQGAP3 AUC was 0.753 (95 % CI 0.651–0.855). AFP AUC was 0.707 (95 % CI 0.604–0.811). CCT3 and IQGAP3 had a larger AUC than AFP had (Fig. 2a, *P* < 0.01). When the cut-off value for CCT3 was selected at 46.5 pg/mL, as determined by maximum sensitivity and specificity, the sensitivity and specificity for CCT3 were 76.6 and 70.5 %, respectively. When the cut-off value for IQGAP3 was selected at 43.5 pg/mL, the sensitivity and specificity for IQGAP3 were 74.5 and 71.6 %, respectively, while the sensitivity and specificity for AFP (20 ng/mL), were 53.2 and 68.2 %, respectively. Thus, CCT3 and IQGAP3 showed superiority to AFP in detecting small HCC tumors.

In addition, plasma CCT3 and IQGAP3 from 38 AFP-negative HCC and 88 cirrhosis patients were analyzed. Their AFP levels were <20 ng/mL. AUC for CCT3 was 0.871 (95 % CI 0.791–0.951, Fig. 2b, *P* < 0.01). When the cut-off value for CCT3 was selected at 46.5 pg/mL, the sensitivity was 92.1 % and specificity was 70.5 %. AUC for IQGAP3 was 0.804 (95 % CI 0.694–0.914, Fig. 2b, *P* < 0.01). When the cut-off value for IQGAP3 was selected at 43.5 pg/mL, the sensitivity was 81.6 % and specificity was 71.6 %.

Furthermore, among 47 patients with small HCC, 27 were negative for AFP. Their AUC for CCT3 was 0.84 (95 % CI 0.735–0.945, Fig. 2c, *P* < 0.01) when differentiating from cirrhosis; its sensitivity and specificity were 88.9 and 70.5 %, respectively, when the cut-off value was selected at 46.5 pg/mL. AUC for IQGAP3 was 0.822 (95 % CI 0.700–0.943, Fig. 2c, *P* < 0.01); when differentiating from cirrhosis, its sensitivity and specificity were 85.2 and 71.6 %, respectively, when the cut-off value was selected at 43.5 pg/mL.

### Combined diagnostic value of plasma CCT3, IQGAP3 and AFP

AUC was calculated to compare the accuracy achieved when using AFP, CCT3 and IQGAP3 for diagnosis of HCC (Fig. 2d). AUC for AFP (0.815, 95 % CI 0.758–0.872) and CCT3 (0.846, 95 % CI 0.791–0.901) was higher than for IQGAP3 (0.808, 95 % CI 0.747–0.869). High levels of plasma CCT3 protein were detected in HCC, and CCT3 had higher sensitivity (87.3 %) than AFP (69.8 %) in differentiating HCC from cirrhosis when the cut-off value was selected at 46.5 pg/mL and 20 ng/mL, respectively (*P* < 0.05). We also analyzed the complementary properties of using CCT3 and IQGAP3 in combination with AFP for the diagnosis of HCC, using a logistic regression model,with “0” represents the cirrhosis group, “1” represents liver cancer. Obtain the following regression equation: P = 1/[1 + e^−(−3.413+0.007X1+0.020X2+0.007X3)^], X1, X2 and X3 respectively represents AFP, CCT3 and IQGAP3, values of OR were: 1.008, 1.020 and 1.007, respectively. 95 % Confidence intervals were (1.007–1.012), (1.010–1.031), (1.001–1.012), respectively (Table 3). Thus, CCT3, IQGAP3 and AFP are the independent factors of HCC. High risk of HCC in patients with high expression of CCT3 is 1.200 times that of lower levels, AFP is 1.008 times and IQGAP3 is 1.007 times, respectively. Thus, the expression of CCT3 and IQGAP3 are independent of AFP.

**Table 3:** Logistic regression analysis

Factor	Regression coefficient	*P*	OR	95 % CI
AFP	0.007	0.001	1.008	1.007–1.012
CCT3	0.020	0.000	1.020	1.010–1.031
IQGAP3	0.007	0.000	1.007	1.001–1.012
Constant	−3.413	0.000	0.033	

The combined use of AFP, CCT3 and IQGAP3 further increased the AUC (0.954; 95 % CI 0.925–0.982), which was higher than that just using AFP alone (Fig. 2e *P* < 0.01).

## Discussion

HCC is one of the most severe types of cancer. It has a high incidence and is the third most common cause of cancer mortality, with 696,000 cases worldwide each year [20–22]. The mortality is partly due to unresponsiveness to treatment, and the 5-year survival rate was <5 % after diagnosis [23]. AFP is a commonly used tumor marker for the early screening of primary HCC. However, AFP as a sole indicator of HCC is of limited value. Statistical data confirmed that the diagnostic sensitivity of AFP for small HCC tumors is only 20–40 %. Nearly one-third of early stage, small HCC tumors (<2 cm) cannot be detected using AFP screening [24, 25]. In contrast, the level of serum AFP does not reflect the severity of the patient’s condition, nor effectively assess HCC prognosis.

Eukaryotic CCT3 consists of two identical rings, each with eight different CCT subunits. Through a variety of structural, functional and cell biology methods, interactions between TRiC and its main substrates, actin and tubulin, have been well characterized [26, 27]. The chaperonins are key molecular complexes that are essential in protein folding to produce stable and functionally competent protein conformations [28, 29]. In this study, high levels of plasma CCT3 protein were detected in HCC, and CCT3 had better sensitivity (87.3 %) than AFP (69.8 %) in differentiating HCC from cirrhosis. Furthermore, the AUC for CCT3 (0.865) was larger than that of AFP (0.815), which is consistent with Yokota et al. [30] who reported increased expression of cytosolic chaperonin CCT in human HCC and colonic carcinoma. Significant overexpression of CCT3 in HCC has also been reported by Wong et al. [12].

IQGAPs is a newly discovered protein family and its sequence displays extensive sequence similar to the catalytic domain of RasGAPS, and four IQ motifs located in the N-terminal which can interact with calmodulin, and in mammals there are three homologous IQGAPs 1–3. IQGAP3 is located at 1q22, which is a hotspot for gene amplification in cancer and expressed in liver and intestines and other organs restrictively [31]. DNA amplification at 1q22 is linked with gastroesophageal carcinoma and infiltrating ductal carcinoma of the breast [32, 33]. In this study, we observed higher levels of serum IQGAP3 in patients with HCC than in patients without HCC. Skawran et al. [13] demonstrated that CCT3 and IQGAP3 are upregulated most significantly by gene set enrichment analysis in HCC.

In this work, we found that concentration of serum CCT3 and IQGAP3 in patients with HCC was correlated with etiology, tumor size, number of cancer nodules and child-pugh classification, indicating that they are novel predictors for HCC. Yuan et al. reported that CCT3 has the potential as a new tumor marker for early detection of cholangiocarcinoma [34]. Ying et al. demonstrated that IQGAP3 may contribute to the pathogenesis of lung cancer by modulating GFR-ERK signaling [35].

The prognosis of HCC can be largely improved if it is detected at an early stage. However, detection of early stage and AFP-negative HCC is still difficult clinically, even with the help of advanced imaging technology. Accurate detection of AFP-negative and small HCC tumors can lead to early diagnosis, treatment, and reduced cancer-related mortality. This study showed that CCT3 and IQGAP3 are superior to AFP in predicting HCC prognosis. In AFP-negative HCC group, the sensitivity of CCT3 and IQGAP3 was 92.1 and 81.6 % and specificity was 70.5, 71.6 %, respectively. We also evaluated whether CCT3 and IQGAP3 could be used as markers for the detection of small HCC tumors. Our data showed that serum CCT3 levels in patients with small tumors (≤2 cm) provided a sensitivity of 76.6 % when distinguishing small HCC from cirrhosis at a cut-off value of 46.5 pg/mL. IQGAP3 had sensitivity of 74.5 % when distinguishing small HCC from cirrhosis at a cut-off value of 43.5 pg/mL. AFP had a sensitivity of 74.5 %. In the AFP-negative small HCC group, the sensitivity of CCT3 and IQGAP3 was 88.9 and 85.2 %, respectively. These results suggest that CCT3 and IQGAP3 can be complementary to AFP in the diagnosis of AFP-negative and small HCC.

Combination of CCT3, IQGAP3 and AFP could significantly increase the sensitivity of each agent for HCC diagnosis. However, the specificity is also reduced. The combined use of AFP, CCT3 and IQGAP3 further increased the AUC (0.954; 95 % CI 0.925–0.982), which was higher than that using AFP alone (0.815; 95 %CI 0.758–0.872).

## Conclusions

In summary, our findings indicate that CCT3 and IQGAP3 are novel biomarkers complementary to AFP in HCC diagnosis, whose expression is independent of AFP. This is especially valuable when AFP is negative and HCC is at an early stage. Thus, CCT3 and IQGAP3 should be useful biomarkers, in combination with AFP, to confirm the diagnosis of HCC. Future works are required to explore whether CCT3 and IQGAP3 can also predict patients’ survival and their usefulness in clinical application.

## Abbreviations

CCT3: chaperonin containing TCP1 complex subunit 3
IQGAP3: IQ-motif-containing GTPase-activating protein-3
HCC: hepatocellular carcinoma
ELISA: enzyme linked immunosorbent assay
AFP: α-fetoprotein
ERK: Ras/extracellular signal-regulated kinase
TNM: tumor-node-metastasis
EDTA: ethylene diamine tetraacetie acid
AUC: areas under the ROC curve
